# R406 elicits anti-Warburg effect via Syk-dependent and -independent mechanisms to trigger apoptosis in glioma stem cells

**DOI:** 10.1038/s41419-019-1587-0

**Published:** 2019-05-01

**Authors:** Shuxin Sun, Dongdong Xue, Zhijie Chen, Ying Ou-yang, Ji Zhang, Jialuo Mai, Jiayv Gu, Wanjun Lu, Xincheng Liu, Wenfeng Liu, Longxiang Sheng, Bingzheng Lu, Yuan Lin, Fan Xing, Zhongping Chen, Yonggao Mou, Guangmei Yan, Wenbo Zhu, Ke Sai

**Affiliations:** 1 Department of Neurosurgery/Neuro-oncology, Sun Yat-sen University Cancer Center, Guangzhou, 510060 China; 2 State Key Laboratory of Oncology in South China, Collaborative Innovation Center for Cancer Medicine, Sun Yat-sen University Cancer Center, Guangzhou, 510060 P. R. China; 3 Department of Pharmacology, Zhongshan School of Medicine, Sun Yat-sen University, Guangzhou, 510080 China; 4 Department of Pediatrics, Sun Yat-sen Memorial Hospital, Sun Yat-sen University, Guangzhou, 510120 China

**Keywords:** CNS cancer, Target identification

## Abstract

Given that glioma stem cells (GSCs) play a critical role in the initiation and chemoresistance in glioblastoma multiforme (GBM), targeting GSCs is an attractive strategy to treat GBM. Utilizing an anti-cancer compound library, we identified R406, the active metabolite of a FDA-approved Syk inhibitor for immune thrombocytopenia (ITP), with remarkable cytotoxicity against GSCs but not normal neural stem cells. R406 significantly inhibited neurosphere formation and triggered apoptosis in GSCs. R406 induced a metabolic shift from glycolysis to oxidative phosphorylation (OXPHOS) and subsequently production of excess ROS in GSCs. R406 also diminished tumor growth and efficiently sensitized gliomas to temozolomide in GSC-initiating xenograft mouse models. Mechanistically, the anti-GSC effect of R406 was due to the disruption of Syk/PI3K signaling in Syk-positive GSCs and PI3K/Akt pathway in Syk-negative GSCs respectively. Overall, these findings not only identify R406 as a promising GSC-targeting agent but also reveal the important role of Syk and PI3K pathways in the regulation of energy metabolism in GSCs.

## Introduction

Glioblastoma multiforme (GBM) is one of the most lethal cancers in adults^1^. Conventional treatments including maximal neurosurgical resection, chemoradiation followed by adjuvant chemotherapy with temozolomide (TMZ), and recently emerging tumor treating fields (TTF), offer limited survival benefits. The prognosis still remains dismal for GBM patients, with the overall survival of approximately 21 months^2^. Numerous studies have reported the existence of cellular hierarchy in GBM tissues, in which a self-renewing subpopulation of tumor cells are capable of generating non-tumorigenic progeny and recapitulating the complexity of GBMs^3^. These glioma cells with stem-like characteristics, also termed as glioma stem cells (GSCs), are considered to account for the tumorigenesis and progression of GBMs. As a result, GSCs are ideal target for the development of novel therapies^4,5^.

Addiction to aerobic glycolysis, namely Warburg effect, is a hallmark of gliomas, by which glioma cells metabolize glucose into lactate for energy generation even under normoxic condition^6^. Glycolysis confers a constant supply of substrates to fuel proliferative glioma cells and provide for an acidic microenvironment in favor of invasion as well as immunosuppression^7,8^. Glycolysis is also critical for the maintenance of GSCs. It has been reported that GSCs are enriched in a hypoxic niche and preferentially rely on glycolysis^9,10^. Reverse of Warburg effect efficiently drives the differentiation of CSCs into non-stem cells and delays tumor growth^11^. Remodeling the energy metabolism is therefore a promising strategy for the management of gliomas.

Spleen tyrosine kinase (Syk) is a member of the Syk/ZAP-70 family of non-receptor protein tyrosine kinases, coupling with a variety of cell surface receptors including Fc receptors, integrins and complement receptors^12^. The expression of Syk is most frequently found in hematopoietic lineage and has been known to play an essential role in the regulation of immune responses and inflammation^13^. Besides, Syk-mediated signaling pathways are involved in other biological functions, such as cellular proliferation, vascular development and adhesion^14^. Syk possesses a schizophrenic reputation in tumorigenesis. In lymphomas derived from B-lineage cells, constitutively activated Syk is responsible for the transformation of normal pre-B cells and provides pro-survival signals by promoting proliferation and blocking apoptosis^15^. On the contrary, Syk can act as a tumor suppressor in many epithelial cell-derived cancers. Decreased expression of Syk resulted from epigenetic silencing are identified in colon cancer, melanoma, nasopharyngeal cancer and hepatocellular carcinoma^16^. Several lines of evidence to support that Syk may play a pro-oncogenic role in glioma. Firstly, transcript levels of Syk are up-regulated in GBM when compared with normal brain samples according to data sets including TCGA and REMBRANDT. Increased expression of Syk is enriched in patients with GBM of mesenchymal subtype and associated with poor prognosis^17^. Secondly, overexpressed Syk is critical for the proliferation and migration of glioma cells. Blockade of Syk in glioma leads to the cell cycle arrest and weakened invasion. Syk inhibitor delays tumor growth and prolongs survival of tumor-bearing mice. Moreover, when pre-treated with of Syk inhibitor, GSCs form spheres with a decreased size in vitro and initiated smaller tumors in vivo^18^. However, the molecular mechanisms underlying the anti-glioma effects of Syk inhibitor still remain evasive.

PI3K/Akt signaling is one of the most frequently dysregulated pathways in human cancers. The aberrantly activated PI3K/Akt pathway contributes to the disturbance in various key cellular processes, such as proliferation, motility, angiogenesis and dissemination of cancer cells^19^. Moreover, PI3K/Akt signaling plays an instrumental role in maintenance of “stemness” and promoting multipotency of cancer stem cells (CSCs). In breast cancer, mutated PI3KCA gene reprograms normally lineage-restricted cancer cells into CSCs at the early stage of tumor initiation, which fulfills these cells with multipotency and results in the intratumoural heterogeneity^20^. Preferential activation of PI3K/Akt pathway via PTEN loss transforms proliferating medulloblastoma cells into CSCs with non-proliferating extensive nodularity morphology and protects these cells from radiation-induced apoptosis^21^.

In the current study, we initiated an exploration of small molecules against GSCs from a compound library consisting of 349 inhibitors. We identified R406, which is the active metabolite of a FDA-approved Syk inhibitor for immune thrombocytopenia (ITP), to effectively reverse glycolysis and trigger apoptosis in GSCs. R406 also synergized with TMZ both in vitro and in vivo. Intriguingly, R406 was effective to eliminate not only GSCs with Syk expression but those negative for Syk. Mechanistically, R406 elicited metabolic shift toward OXPHOS and induced ROS accumulation via disruption of either Syk/PI3K pathway in Syk-positive GSCs or PI3K/Akt signaling in Syk-negative GSCs respectively. Overall, our study provides evidence that R406 is a promising therapeutic agent for the treatment of patients with GBM by targeting GSCs, and Syk as well as PI3K/Akt signaling play critical role in the metabolism and survival of GSCs.

## Results

### Drug screening identifies R406 as an effective inhibitor against GSCs

**Fig. 1:**
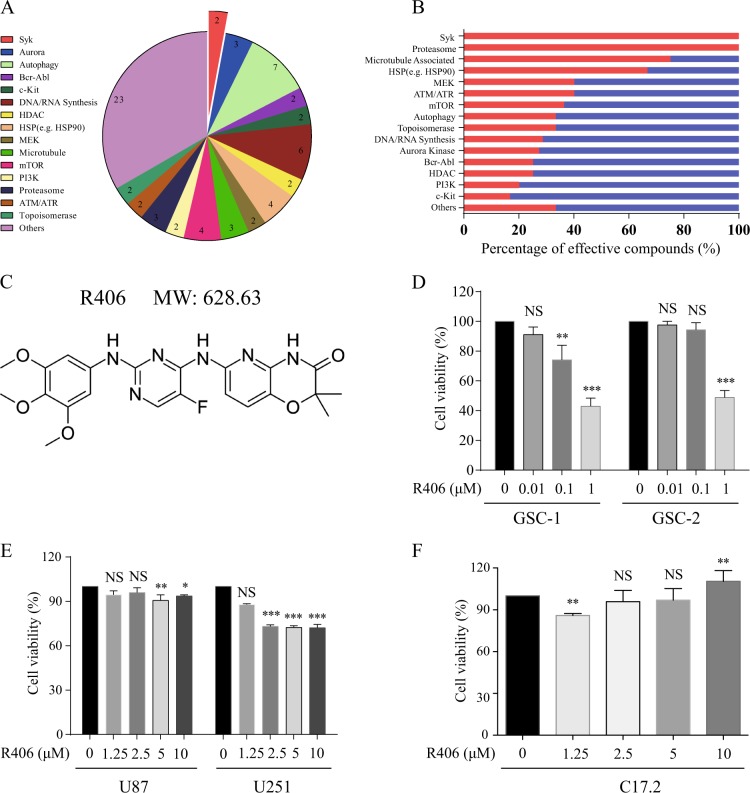
R406 exhibits potent activities against GSCs. **a** A total of 69 inhibitors were identified from a library of 349 chemical compounds to be effective (IC50 < 1 μM) against GSC-1 cells. **b** The red and blue bars represented the percentage of effective and less effective inhibitors for the same target, respectively. **c** Chemical structure of R406. **d** Incubation with R406 significantly reduced the cell viability of both GSC lines, with an IC50 of 0.75 μM for GSC-1 and 0.89 μM for GSC-2 respectively. **e** U87 and U251 were insensitive to R406, with a calculated IC50 of more than 1 mM for both cell lines. **f** C17.2 mouse neural progenitors were not susceptible to R406. R406 at the concentration as high as 10 μM was non-toxic to C17.2 cells. (*MW* molecular weight; *NS* not significant; *p *<* 0.05; **p *<* 0.01; ***p *<* 0.001*, statistical difference compared with the control*.)

To explore potential candidate inhibitors against GSCs, we performed the drug screening in a library of 349 chemical compounds with GSC-1 cells. Inhibitors with an IC50 less than 1 μM were documented and categorized according to their molecular targets. A total of 69 inhibitors were identified, as summerized in Fig. 1a and b. Among them, R406 (Fig. 1c) and its prodrug fostamatinib, which are Syk inhibitors, were selected for further analysis because no evidence in the literatures has been found to show their effects on GSCs. To verify the anti-GSC effect, we treated another GSC cell line, GSC-2, with R406. We found R406 significantly inhibited the proliferation of GSC-2 cells, with an IC50 of 0.89 μM (Fig. 1d). However, non-GSC glioma cell lines seemed far more insensitive to R406. The treatment of the established U87 and U251 cells with R406 at a concentration of 10 μM only resulted in a less than 20% reduction in cell viability (Fig. 1e). In fact, the estimated IC50 of R406 were more than 1 mM for U87 and U251. Moreover, in order to determine the effect of R406 on normal neural stem cells, we incubated the inhibitor with C17.2 cells, which were immortalized mouse neural progenitors. Viability analysis demonstrated that R406 was not toxic to C17.2 cells, even at the concentration of 10 μM (Fig. 1f).

### R406 impairs self-renewal and induces apoptotic death in GSCs

**Fig. 2:**
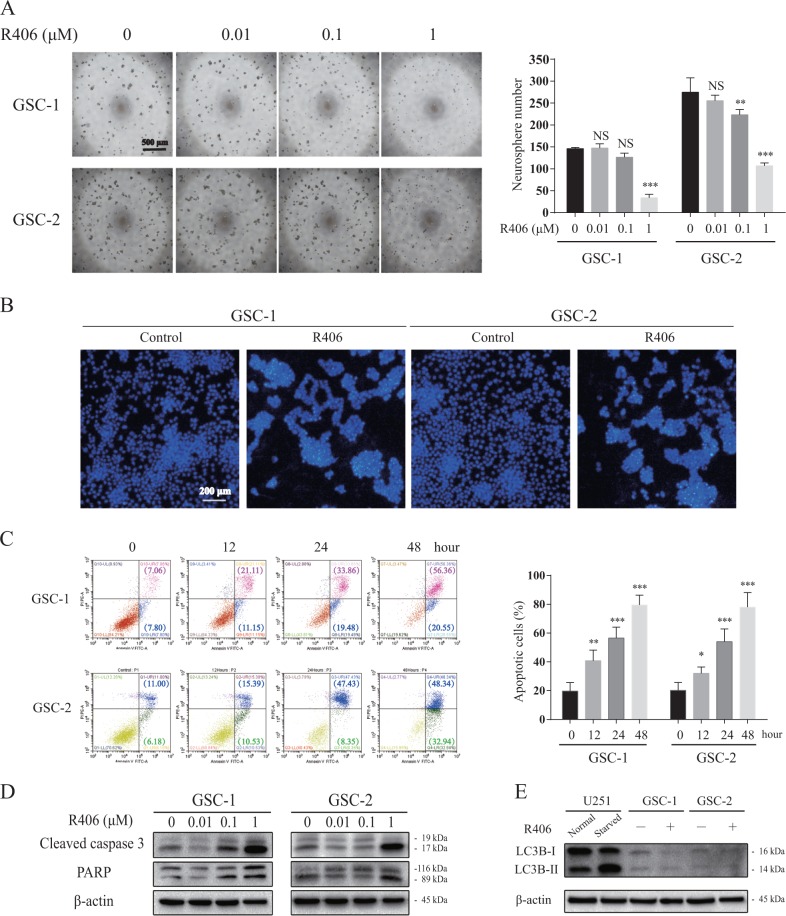
R406 inhibits neurosphere formation and induces apoptotic death in GSCs. **a** Representative images of neurosphere formation of GSC-1 and GSC-2 treated with different concentration of R406 for 72 h. Quantification of neurosphere number showed R406 at a concentration of 1 μM for 72 h effectively impaired neurosphere formation of GSC-1 and GSC-2. **b** Hoechst 33342 staining demonstrated that R406 treatment led to chromatin condensation in GSCs. **c** Analysis with Annexin V showed that R406 cause an increase in the percentage of apoptotic cells in GSCs, in a time-dependent manner. **d** Activated caspase 3 and cleaved PARP induced by R406 were measured by immunoblotting analysis. **e** Western blot demonstrated that no changes in protein level of LC3B in GSCs after R406. Starved U251 cells were positive control for LC3B expression. (*NS* not significant; *p *<* 0.05; **p *<* 0.01; ***p *<* 0.001, *statistical difference compared to the control*.)

To determine the effect of R406 on GSCs, we initiated with the neurosphere assay, which provides an in vitro assay that can be utilized to evaluate the capacity for self-renewal of multiple stem cell types. Neurosphere assay demonstrated that R406 incubation resulted in a dose-dependent reduction of neurosphere formation (Fig. 2a). More than 50% reduction in number of neurospheres was observed when GSC-1 and GSC-2 cells were exposed to R406 at a concentration of 1 μM, suggesting R406 impairs the self-renewal of GSCs (Fig. 2a), whereas R406 had no influence on the colony formation of C17.2 normal neural stem cells (data not shown). Given that R406 is capable of triggering apoptosis in chronic lymphocytic leukemia and multiple myeloma^22,23^, we then investigated whether R406 eliminates GSCs through apoptotic death. We firstly stained GSCs with Hoechst 33342 to detect morphological changes of nuclei of GSCs after R406 incubation. We found that treatment with R406 resulted in chromatin condensation and nuclear fragmentation in GSCs (Fig. 2b), implying the induction of apoptosis. Quantitative analysis with flow cytometry demonstrated that R406 induced time-dependent apoptosis in GSCs. Exposure to R406 at a concentration of 1 μM for 48 h resulted in a more than 4-fold increase in Annexin V positive cells in both GSC-1 and GSC-2, which was statistically significant (Fig. 2c). The activation of caspase 3 and the following cleavage of PAPR shown by western blot confirmed the initiation of apoptosis by R406 (Fig. 2d). In addition, to rule out the possibility that autophagy is involved in the anti-GSC effect of R406, we tested the protein level of LC3, a marker for autophagy. No changes in the expression of LC3 were detected (Fig. 2e).

### R406 triggers metabolic shift from glycolysis towards oxidative OXPHOS in GSCs

**Fig. 3:**
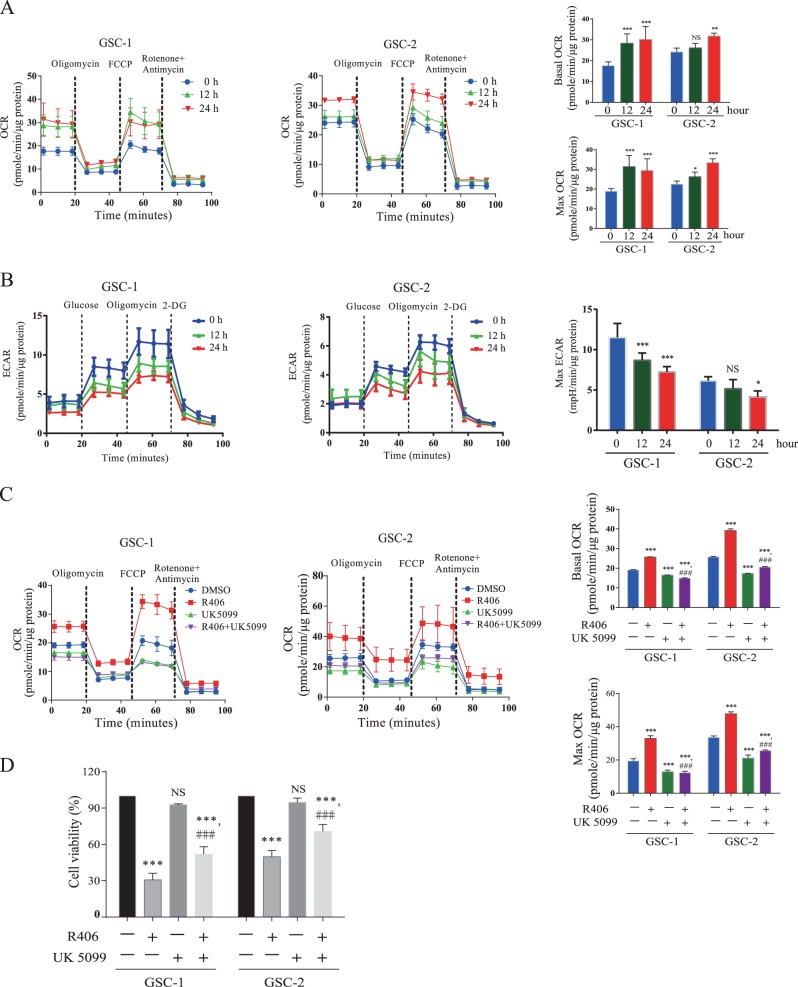
R406 treatment results in the decreased glycolysis and enhancement of OXPHOS in GSCs. **a** Time-dependent effect of R406 treatment on the OCR in GSCs. GSC-1 and GSC-2 cells were incubated with 1 μM for the indicated time and the OCR was monitored by using Seahorse Bioscience extracellular flux analyzer in real time. Dotted lines represent treatment of cells with the indicated compounds. R406 treatment significantly elevated basal and maximal OCR in GSCs. **b** R406 significantly attenuated ECAR in GSCs. **c** Addition of UK5099, an OXPHOS inhibitor, suppressed the elevation of OCR induced by R406 in GSCs. **d** Inhibition of OXPHOS with UK5099 partially rescued cell death caused by R406 in GSCs. (*NS* not significant; *p *<* 0.05; **p *<* 0.01; ***p *<* 0.001, *statistical difference compared to the control*. ^*###*^*p* *<* 0.001, *statistical difference compared to the group treated with R406*.)

As reported previously, the status of energy metabolism is pivotal for GSC maintenance^24^. We therefore asked whether R406 influences the balance of glycolysis and OXPHOS in GSCs. By using Seahorse Bioscience extracellular flux analyzer, we found a significant increase in the basal and maximal oxygen consumption rate (OCR) in GSCs after 24- and 48-hour incubation of R406 compared with the control (Fig. 3a), indicating the enhanced OXPHOS. In addition, we also demonstrated that R406 diminished the extracellular acidification rate (ECAR) in GSCs (Fig. 3b). These results elucidated that R406 induced an anti-Warburg effect in GSCs. To better understand the consequence of this metabolic shift, we treated GSCs with a potent mitochondrial pyruvate carrier inhibitor UK5099, which promotes glycolysis by antagonizing pyruvate-driven respiration. Incubation of UK5099 at a concentration of 2 μM for 48 h significantly weakened the transition towards OXPHOS induced by R406, which is reflected by the decreased basal and maximal OCR (Fig. 3c). The repressed mitochondrial oxidative respiration protected GSCs from cell death caused by R406 (Fig. 3d). Taken together, these data clearly supported that R406 treatment leads to metabolic transition towards OXPHOS, which is lethal for GSCs.

### Production of ROS by the enhanced OXPHOS is responsible for R406-induced apoptosis

**Fig. 4:**
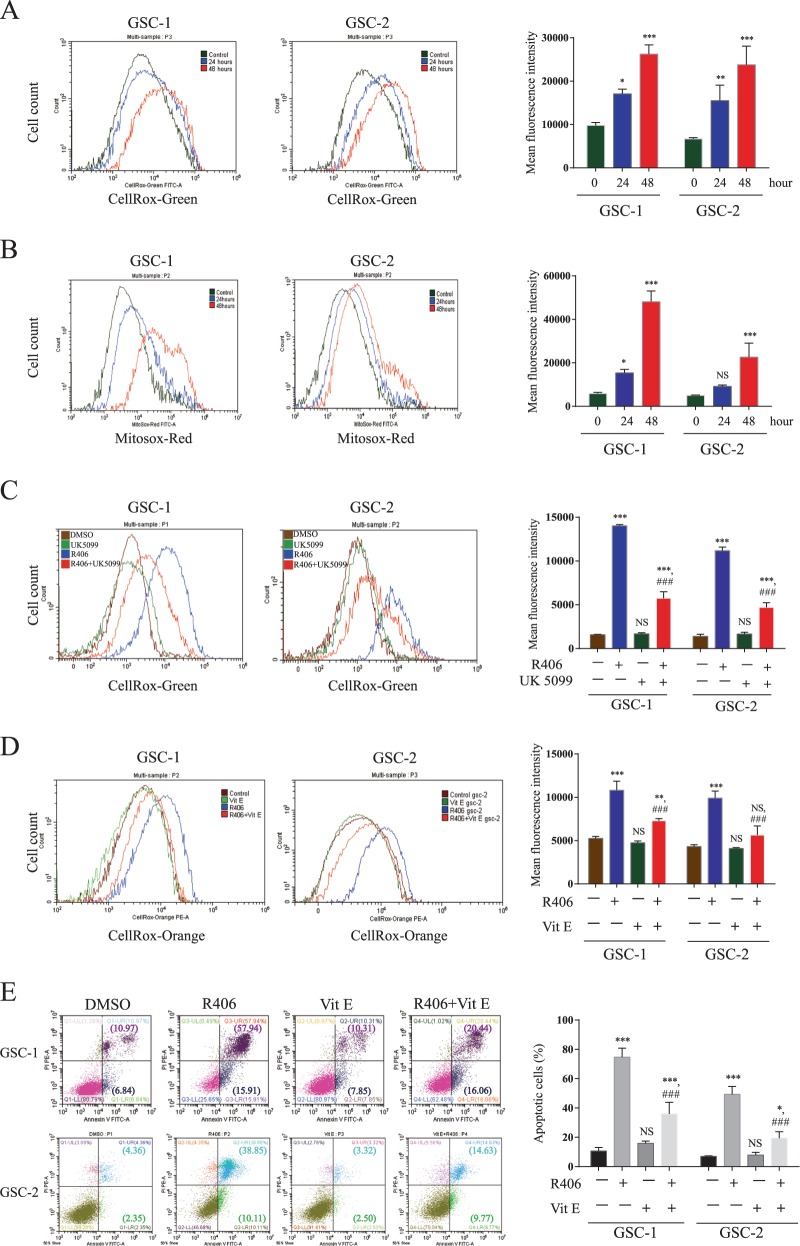
Excess ROS generated by OXPHOS mediates apoptosis induced by R406 in GSCs. **a** ROS level after R406 treatment at different time point was measured by flow cytometry with CellROX green probe. **b** MitoSOX red dye was used to detect the changes of mitochondrial ROS in GSCs after the incubation with R406. **c** Inhibition of OXPHOS by UK5099 efficiently attenuated the production of ROS resulted from R406 treatment in GSC. **d** Vitamin E, a ROS scavenger, significantly reduced ROS level in GSCs treated with R406. **e** Addition of Vitamin E attenuated apoptosis induced by R406. (*NS* not significant; *p *<* 0.05; **p *<* *0*.01; ***p *<* 0.001, *statistical difference compared to the control*. ^*###*^*p* *<* 0.001, *statistical difference compared to the group treated with R406*.)

Excess cellular levels of reactive oxygen species (ROS) cause oxidative stress and trigger programmed cell death such as apoptosis^25^, and we therefore hypothesized that ROS accumulation might be the connection between the metabolic shift and R406-induced apoptosis. We firstly applied the fluorescent probe CellROX green to quantify the ROS level in R406-treated GSCs. We found that ROS concentration substantially increased as compared with control cells 48 h after R406 addition (Fig. 4a). Intracellular ROS are mainly generated in mitochondria but can also be produced as a result of protein folding-related oxidation-redox reaction in endoplasmic reticulum^26,27^. To distinguish the organelles where ROS is produced after R406 treatment, we measured the generation of ROS in GSCs by using MitoSOX red mitochondrial superoxide indicator, which is a fluorogenic dye specifically targeted to mitochondria. Flow cytometry showed that R406 significantly shifted histograms of MitoSOX fluorescence to the right in a time-dependent manner (Fig. 4b), indicating an increase in mitochondrial ROS production in GSCs. To further determine whether the increased ROS level is attributed to the enhanced OXPHOS, we co-treated GSCs with R406 and UK5099. Inhibition of OXPHOS by UK5099 significantly attenuated the production of ROS (Fig. 4c). Next, in order to clarified the role of ROS in R406-induced apoptosis, GSC-1 and GSC-2 cells were exposed to the ROS scavenger Vitamin E. GSCs were incubated with 1 μM R406 in the absence or presence of 50 μM Vitamin E for 48 h. As shown in Fig. 4d, e, ROS level declined significantly and apoptosis resulted from R406 was abated by Vitamin E, suggesting that ROS is essential for the cytotoxicity of R406.

### Suppression of Syk/PI3K signaling mediates R406-induced OXPHOS enhancement and subsequent ROS-dependent apoptosis in Syk-positive GSCs

**Fig. 5:**
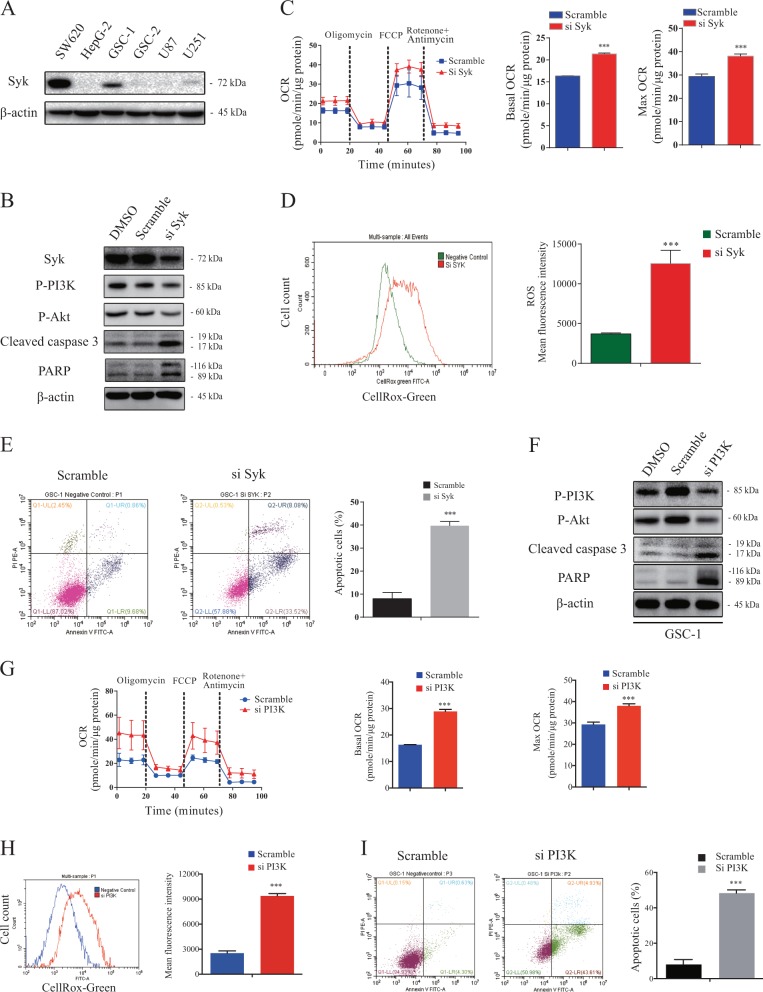
R406 targets Syk/PI3K pathway against Syk-positive GSCs. **a** Western blot demonstrated that Syk is highly expressed in GSC-1 but not GSC-2, U87 or U251. Protein extracted from non-glioma tumor cell line SW620 and HepG-2 were positive and negative control for Syk expression respectively. **b** Syk-specific siRNA decreased Syk expression and inhibited the phosphorylation of PI3K and Akt, which resulted in the activation of caspase 3 and the cleavage of PARP in GSC-1. **c** Knock-down of Syk expression elevated basal and maximal OCR in GSC-1. **d** Depletion of Syk promoted the production of ROS in GSC-1. **e** Depletion of Syk using specific siRNA resulted in a substantial increase in the percentage of apoptotic cells in GSC-1. **f** Transient knock-down of PI3K inhibited the phosphorylation of PI3K and Akt, and triggered caspase activation and PARP cleavage in GSC-1. **g** Silencing PI3K increased basal and maximal OCR in GSC-1. **h** ROS accumulated after depletion of PI3K in GSC-1. **i** Knock-down of PI3K significantly induced apoptosis in GSC-1. (***p *<* 0.001, *statistical difference compared to the scramble group*.)

Given that R406 is developed as a Syk inhibitor, we then sought to determine whether R406 exhibited its anti-GSC efficacy through blocking Syk pathway. To this end, we investigated the Syk expression in GSCs and two non-GSC glioma cell lines. Western blot revealed that Syk was up-regulated in GSC-1 but not in GSC-2, U87 or U251 (Fig. 5a). In the Syk-positive GSC-1 cell line, we knocked down Syk with transient RNAi. Western blot demonstrated that the specific siRNA decreased the protein expression of Syk and inhibited the phosphorylation of PI3K and Akt, which are the down-stream target of Syk pathway (Fig. 5b). Depletion of Syk robustly promoted OXPHOS by increasing the basal and maximal OCR (Fig. 5c), and remarkably elevated the level of intracellular ROS (Fig. 5d). In addition, down-regulation of Syk activated caspase cascade (Fig. 5b) and induced apoptotic death, by increasing the percentage of apoptosis from 10.5 to 41.6% in GSC-1 (Fig. 5e). Next, to answer the question whether the downstream PI3K mediated the anti-GSC effect of inhibited Syk pathway, we also suppressed the expression of PI3K using siRNA (Fig. 5f). Direct inhibition of PI3K resulted in OXPHOS enhancement (Fig. 5g) and subsequent ROS-mediated apoptosis in GSC-1 cells (Fig. 5g, i), which is similar to the anti-GSC effect of Syk knock-down and R406 treatment. Collectively, these data indicated that R406 elicits energy shift as well as ROS-induced apoptosis by suppressing Syk/PI3K pathway in Syk-positive GSCs.

### Prohibition of PI3K/Akt pathway by R406 contributes to the energy shift towards OXPHOS in Syk-negative GSCs

**Fig. 6:**
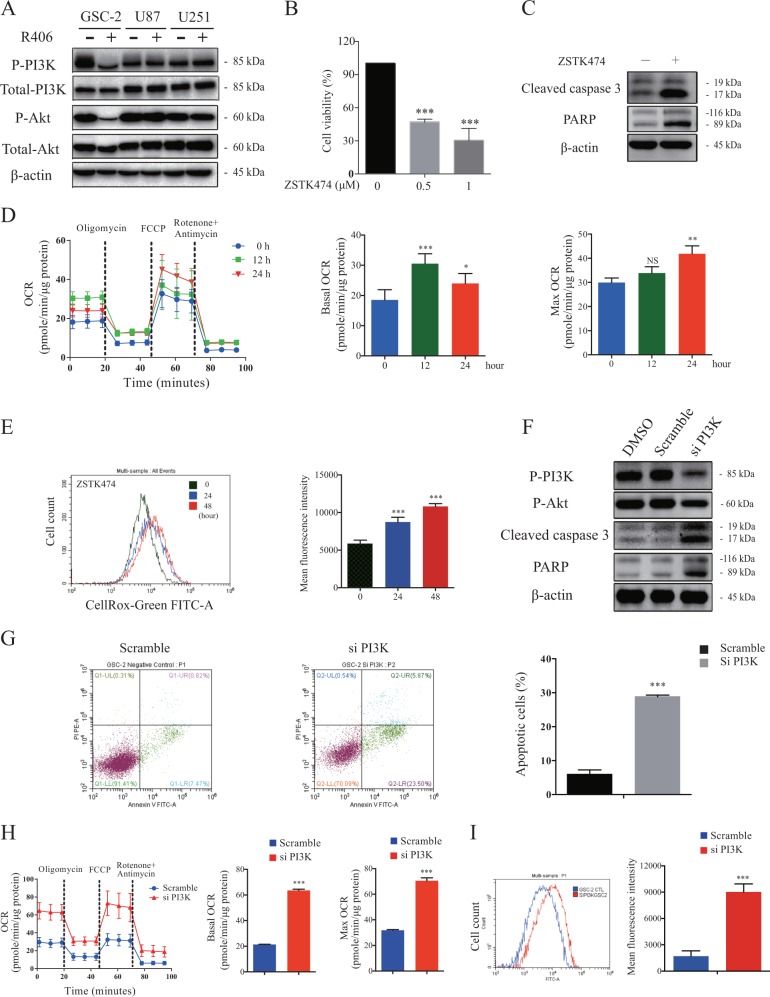
R406 exhibits anti-GSC effect via suppressing PI3K/Akt pathway in Syk-negative GSCs. **a** R406 significantly suppressed the phosphorylation of PI3K and Akt in GSC-2 but not in U87 and U251. **b** The PI3K/Akt pathway inhibitor, ZSTK474, eliminated GSCs in a dose-dependent manner. **c** Western blot demonstrated ZSTK474 activated caspase 3 and caused the cleavage of PARP in GSCs. **d** Basal and maximal OCR was elevated when GSCs were incubated with ZSTK474 at the indicated time point. **e** Flow cytometry for CellRox staining showed that treatment with ZSTK474 promoted ROS production in a time-dependent manner. **f** Western blot showed that the knock-down of PI3K resulted in decreased phosphorylation of PI3K and Akt as well as was the caspase activation and PARP cleavage in GSC-2. **g** Depletion of PI3K induced apoptotic death in GSC-2. **h** Increased basal and maximal OCR were observed when PI3K was silenced using specific siRNA. **i** Knock-down of PI3K promoted ROS production in GSC-2. (***p *<* 0.001, *statistical difference compared to the scramble group*.)

Although negative for Syk expression (Fig. 5a), GSC-2 cells displayed distinct sensitivity to R406. We thus speculated that pathways other than Syk are responsible for the effects of R406 on GSC-2. As we have shown in GSC-1, inhibition of PI3K/Akt pathway by depleting PI3K expression mimicked the anti-GSC effect of R406. To establish the link between R406 treatment and PI3K/Akt pathway in GSC-2, we firstly determined the phosphorylation of PI3K and Akt in GSC-2 and non-GSC glioma cell lines. Western blot demonstrated that incubation of R406 at a concentration of 1 μM for 48 h substantially inhibited the phosphorylation of PI3K and Akt in GSC-2 but not in U87 and U251 cells (Fig. 6a), implying that R406 could execute the anti-GSC activity through targeting PI3K/Akt pathway. Next, to test the response to PI3K/Akt inhibition, we treated GSC-2 cells with a specific inhibitor ZSTK474. As shown in Fig. 6b, c, ZSTK474 efficiently reduced cell viability by inducing apoptosis in GSC-2. In addition, suppression of PI3K/Akt signaling with ZSTK474 resulted in remarkable enhanced OXPHOS (Fig. 6d) and led to an accumulation of intracellular ROS (Fig. 6e) in a time-dependent manner, which is comparable to the effects of R406. Furthermore, to confirm the critical role of PI3K/Akt in GSC-2, we silenced PI3K using siRNA (Fig. 6f). Similarly, knock-down of PI3K triggered apoptosis (Fig. 6g) by activing caspase 3 followed by the cleavage of PARP in GSC-2 (Fig. 6f). Compared with the control group, GSC-2 cells with depleted PI3K presented with a significantly elevated OCR (Fig. 6h) and enormous levels of intracellular ROS (Fig. 6i). Together, these findings suggested that the anti-GSC effects of R406 is attributed to the disruption of PI3K/Akt signaling in Syk-negative GSCs.

### R406 sensitizes GSCs to TMZ both in vitro and in vivo

**Fig. 7:**
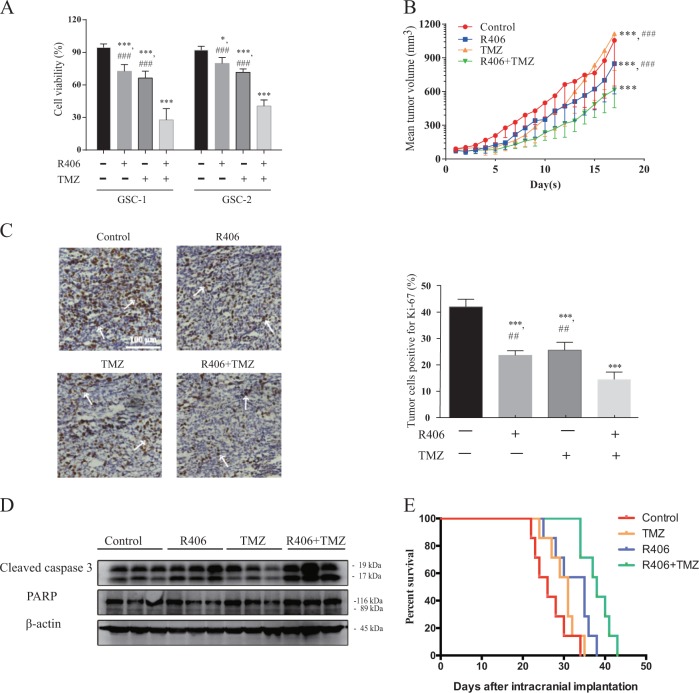
R406 synergistically enhances TMZ cytotoxicity against GSCs in vitro and in vivo. **a** Cell viability of GSCs after treatment with either R406 alone, TMZ alone or combination of two agents was measured using Muse cell analyzer. Quantification analysis demonstrated that combination of R406 and TMZ was superior to monotherapy with the single agent. **b** Combination of R406 and TMZ significantly delayed subcutaneous tumor growth in vivo compared to monotherapy. **c** IHC for tumor sections demonstrated that addition of R406 to TMZ decreased the numbers of proliferative cells, which were positive for Ki-67 (white arrow). **d** Protein was extracted from xenograft specimens and allocated for Western blot. Enhanced activation of caspase 3 was found in mice treated with combination of R406 and TMZ. **e** R406 plus TMZ significantly prolonged the survival of mice in the orthotopic model compared to monotherapy either with R406 or TMZ alone. (*NS* not significant; *p *<* 0.05; ***p *<* 0.001, *statistical difference compared to the control*. ^##^*p* *<* 0.01, ^###^*p* < 0.001, statistical difference compared to the group treated with the combination of R406 and TMZ.)

Temozolomide (TMZ), an DNA-alkylating agent, is the standard-of-care chemotherapeutic drug for GBM. However, substantial resistance is frequently observed in clinical practice. We thus asked whether R406 could enhance the efficacy of TMZ against GSCs. To this end, we evaluated cell viability after treating GSCs with either R406, TMZ, or combination of R406 and TMZ respectively. Monotherapy with either R406 or TMZ moderately decreased the viability of GSCs, whereas the combined treatment resulted in a dramatic increase in cell death of GSCs (Fig. 7a). The calculated combination index (CI) was 0.262 for GSC-1 and 0.280 for GSC-2 respectively. The CI of less than 1 for both GSCs indicated a synergistic effect of R406 and TMZ.

Our in vitro results strongly suggested there could be a benefit for addition of R406 to TMZ for GBM treatment in vivo. We firstly assessed this possibility by implanting GSCs subcutaneously into immunocompromised mice. After the establishment of tumors, mice were administered with either vehicle, R406 or TMZ alone, or R406 and TMZ in combination respectively. As shown in Fig. 7b, c, GSCs efficiently initiated gliomas in the animal model. Treatment with TMZ alone was effective at the early stage but failed to retard tumor growth lately. The tumor proliferated with an accelerated speed and the tumor size surpassed that in the control group 15 days after implantation. The rebounded growth of glioma in vivo is similar to the recurrence observed in GBM patients. Monotherapy with R406 was superior to delay the tumor growth compared to that with TMZ alone. Notably, a stable and significant reduction in tumor size was found in mice received with the combination of R406 and TMZ compared to the control group and monotherapy with either R406 or TMZ alone. Analysis of tumor sections from treated animal demonstrated that cell proliferation (Ki-67) decreased with the addition of R406 to TMZ therapy (Fig. 7c). Enhanced apoptosis was identified in the group with combined treatment, which was unveiled by the prominent activation of caspase 3 in xenograft tissues (Fig. 7d). Because the microenvironment of brain strongly impacts the progression of gliomas and the brain-blood barrier (BBB) significantly influences the entry of therapeutic agents into CNS, we then established the orthotopic animal model to determine the efficacy of the combined therapy against GSCs. Similarly, we found that the R406 plus TMZ was superior to prolong the survival of tumor-bearing mice compared to either R406 (*p* < 0.05) or TMZ (*p* < 0.01) alone. These data collectively suggested that R406 attenuated the resistance of GSCs to TMZ in vitro and was synergistic with TMZ to inhibit GSC-initiated tumor by promoting apoptosis in vivo.

## Discussion

Despite recent advances in therapy, GBM is still one of the most devastating cancers. The development of novel treatment for GBM is of urgent necessity. In the current study, we screened from an anti-cancer compound library and identified R406 as potent agent against GSCs, which play pivotal role in tumorigenesis and treatment resistance of glioma. To the best of our knowledge, this is the first study to display the efficacy of R406 for the treatment of glioma stem cells.

R406 and its prodrug fostamatinib are promising Syk inhibitors for the treatment of several human diseases, such as allergy, autoimmunity and malignancies^14^. R406 alleviates the severity of autoimmune arthritis in mice and provides clinical benefit in patients with rheumatoid arthritis^28^^,29^. Administration of R406 also demonstrates efficacy for autoantibody-induced thrombocytopenia in animal models, and autoimmune thrombocytopenic purpura in humans^30^. In addition, deactivation of Syk signaling by R406 not only induces apoptosis in chronic lymphocytic leukemia cells but also inhibits protective stroma signals^31^. In the current study, we found that Syk and its downstream target PI3K enhanced the self-renewal and antagonized apoptosis through promoting glycolysis and the inhibition of ROS production in Syk-positive GSCs. R406 reversed the Warburg effect by significantly deactivating Syk/PI3K pathway and efficiently eliminating GSCs both in vitro and in vivo. Our present study revealed that Syk was a pro-survival signal for GSCs and could be efficiently targeted by R406.

R406 is also endowed with ability to eliminate GSCs via pathways other than Syk. GSC-2, another GSC line we utilized in this study, was undetectable for Syk protein but exhibited distinct susceptibility towards R406. The finding that incubation of R406 caused significant prohibition of PI3K/Akt signaling in GSC-2 inspired us to investigate the connection between PI3K/Akt suppression and the anti-GSC effect of R406. Our study demonstrated that R406 dramatically down-regulated the phosphorylation of key component in PI3K/Akt signaling and enhanced OXPHOS followed by the accumulation of ROS, which sabotaged the population of GSCs by triggering apoptosis. Interference of this oncogenic pathway with either the specific inhibitor ZSTK474 or siRNA silencing recapitulated the anti-GSC effect of R406. These observations confirmed that PI3K/Akt signaling was crucial for GSC maintenance and R406 exerted anti-GSC effects through the inhibition of this oncogenic pathway.

In this study, we clearly demonstrated that R406 elicited the anti-Warburg effect in GSCs. Blockade of glycolytic flux by R406 compromised the proliferation and neurosphere formation of GSCs. The simultaneously increased OXPHOS activity resulted in a significant accumulation of ROS, which is responsible for the apoptotic death in GSCs. Addition of the OXPHOS inhibitor partially reversed ROS-mediated apoptosis of GSCs induced by R406, indicating the critical role of metabolic transition in the anti-GSC effect of R406. Many oncogenes and related protein are involved in the modulation of energy metabolism^32^. Here we revealed that suppression of Syk/PI3K in Syk-positvie GSCs or PI3K/Akt pathways in Syk-negative GSCs by R406 efficiently induced the metabolic switch towards OXPHOS. Our findings provided insight into the pivotal role of Syk/PI3K and PI3K /Akt pathways in the energy metabolism in GSCs.

Temozolomide (TMZ) is the first-line chemotherapeutic agent for GBM. Intrinsic and required resistance to TMZ makes the recurrence inevitable. Strategies to overcome insensitivity are critical to successful treatment of this cancer. R406 was synergistic with TMZ to eliminate GSCs in vitro. Moreover, addition of R406 significantly sensitized GSCs to TMZ by suppressing proliferation and inducing apoptosis in vivo. Inhibition of glycolysis by silencing lactate dehydrogenase has been reported to reverse the resistance to radiation and TMZ in gliomas^33^. The metabolic shift from glycolysis towards OXPHOS triggered by R406 could account for the synergy with TMZ.

Toxicity to the central nervous system is the major concern for the clinical application of inhibitors such as R406 with the potential for the treatment of brain tumors in humans. Several lines of evidence suggest that the neurotoxicity of R406 is minimal. We treated normal neural stem cell line C17.2 with R406 and found that C17.2 cells were not susceptible to R406. At the concentration of 10 μM, R406 had no inhibitory influence on the proliferation of C17.2 cells. R406 also failed to impair the colony formation of C17.2 cells. In addition, fostamatinib, the prodrug of R406, has been investigated clinically in several diseases and approved recently by the U.S. FDA for the treatment of ITP^34^. According to the safety profiles from clinical trials, the most common adverse events of fostamatinib include hypertension, diarrhea, nausea and dizziness. No severe neurotoxicity has been reported^35^.

In conclusion, our study demonstrated that R406 exhibited potent activities against GSCs, which endows this inhibitor with promising potential to treat gliomas. The suppression of Syk/PI3K signaling in Syk-positive GSCs or PI3K/Akt pathway in Syk-negative GSCs by R406 resulted in the metabolic transition towards OXPHOS followed by the accumulation of ROS and subsequently the initiation of apoptosis. R406 was also capable of synergistically enhancing cell killing of TMZ in GSCs. These findings not only provide evidences for the effectiveness of R406 to eliminate both Syk-positive and Syk-negative GSCs, but also reveal that Syk and PI3K/Akt pathways play critical role in the modulation of energy metabolism in GSCs.

## Materials and methods

### Cell culture and reagents

All studies involving human glioma samples and animals were approved by the institutional ethics committee of Sun Yat-sen University Cancer Center, Sun Yat-sen University and Zhongshan School of Medicine, Sun Yat-sen University. GSC-1 and GSC-2 cell line were derived from human GBM tumor samples and were characterized as reported previously^36,37^. GSCs were cultured in serum-free DMEM/F12 medium supplemented with 2% B27 (Gibco), 20 ng/mL basic fibroblast growth factor (bFGF, Gibco), and 10 ng/mL epidermal growth factor (EGF, Gibco). GSC-spheres were dissociated by using StemPro Accutase Cell Dissociation Reagent (Gibco) for serially passaging or further experiments. The human established glioma cell line U87 was purchased from the American Type Culture Collection (ATCC) and U251 was obtained from Shanghai Institute of Cell Biology, the Chinese Academy of Sciences, China. The established glioma cell lines were cultured in DMEM supplemented with 10% fetal bovine serum (FBS) (Gibco). The C17.2 murine neural stem cell line was generously provided by Professor Rongbiao Pi of Department of Pharmacology, Zhongshan School of Medicine, Sun Yat-sen University, China. C17.2 cells were routinely cultured in DMEM supplemented with 10% FBS (Gibco), 5% horse serum (Gibco) and 2 mM L-glutamine. All of these cell lines were maintained in a humidified atmosphere at 37 °C under 5% CO_2_. Reagents including R406 (#S1533), Vitamin E (#S4686), UK5099 (#S5317) and ZSTK474 (#S1072) were purchased from Selleck Chemicals.

### Drug screening for potential effective inhibitors against GSCs

A library of 349 anti-cancer compounds library (#L3000, Selleck Chemicals) was used to identify inhibitors effectively targeting GSCs. Briefly, GSC-1 cells were seeded in 96-well flat-bottomed plates at a density of 4 × 10^3^ in 100 μL conditioned medium per well. Chemical compounds from the library were prepared with gradient dilution and were incubated with GSCs for 48 h. Cell viability was measured by using CCK-8 assay and the IC50 of each compound was calculated. The compounds with an IC50 less than 1 μM were considered as candidates for further analysis.

### Neurosphere formation assay

Neurosphere formation assay was performed as described previously^38^. Briefly, single GSCs were seeded in 96-well flat-bottomed plates at a density of 4 × 10^3^ cells per well. GSCs were treated with DMSO (controls) or R406 at the concentration of 0.01–1 μM. After 72 h incubation at 37 °C in a humidified 5% CO_2_ atmosphere, the number of neurospheres were quantified under a microscope.

### Cell viability assay

Cell Counting Kit 8 (CCK-8, #CK04, Dojindo Laboratories) was employed to determine cell viability after R406 treatment. Briefly, in case of GSCs, triturated cells were seeded in 96-well flat-bottomed plates at a density of 4 × 10^3^ in 100 µL conditioned medium per well and incubated with DMSO or 0.01–1 μM of R406. For U87 and U251, 2 × 10^3^ cells in 100 µL DMEM supplemented with 10% FBS were seeded in each well and exposed to DMSO or R406 at the concentration of 1.25–10 μM. After 48-hour incubation at 37 °C in a humidified 5% CO_2_ atmosphere, 10 µL CCK-8 solution was added to each well and absorbance at a wavelength of 450 nm was quantified. To test the synergy of R406 and TMZ, cell viability was determined using the Muse Count & Viability Reagent (Merck KGaA, Darmstadt, Germany) according to the manufacturer’s instruction.

### Hoechst 33342 staining

Hoechst staining was employed to determine the nuclear condensation of the apoptotic cells. Briefly, triturated GSCs were seeded in 24-well flat-bottomed plates at a density of 1 × 10^5^ in 500 µL conditioned medium per well and were incubated with DMSO or R406 at the concentration of 1 µM for 48 h. The treated GSCs were then stained with Hoechst 33342 at a concentration of 5 µg/mL for 20 min. The stained nucleic DNA was visualized under a fluorescence microscope.

### Annexin V analysis for apoptosis

In order to determine apoptosis, Annexin V/PI kit (#V13242, Invitrogen) was applied according to the manufacturer’s instruction. Briefly, treated cells were washed twice with cold PBS and then resuspended in binding buffer. Annexin V and PI were added. After gentle vertex and incubation in darkness at room temperature for 15 min. The extent of apoptosis in the samples was measured with BD FACS Calibur flow cytometer.

### Detection of ROS generation

Intracellular ROS production was determined by CellROX Green or Orange Flow Cytometry Assay Kit (#C10492, Invitrogen) and MitoSOX Red Mitochondrial Superoxide Indicator (#M36008, Invitrogen) according to the manufacturer’s instruction. Briefly, for CellROX assay, treated GSCs were incubated with 5 μM CellROX dye at room temperature for 2 h. Quantification was through determining the mean fluorescence intensity by flow cytometry using 488-nm laser for CellROX green (508/525 nm, Excitation/Emission peaks) and for CellROX orange (545/565 nm) respectively. For MitoSOX analysis, cells were incubated with 5 μM of MitoSOX reagent for 20 min at 37 °C after which fluorescence was measured at a wavelength of 610 nm using flow cytometry.

### Western Blot

Soluble proteins from treated cells or xenograft tissues were harvested and lysed in solution containing 50 mM HEPES (pH 7), 150 mM NaCl, 1.5 mM MgCl_2_, 1 mM EGTA, 100 mM NaF, 10 mM sodium pyrophosphate, 10% glycerol, 1% Triton X-100, 1 mM Na_3_VO_4_, 10 µM pepstatin, 10 µg/ml aprotinin, 5 mM iodoacetic acid, and 2 µg/mL leupeptin. Equal amounts of protein were resolved by SDS-PAGE and transferred to polyvi-nylidene difluoride membranes (Roche). The membranes were then probed with the following primary antibodies: Cleaved caspase 3 (#9664S, Cell Signaling Technology), PARP (#9532S, Cell Signaling Technology), Syk (#13198S, Cell Signaling Technology), PI3K (#4257S, Cell Signaling Technology), Tyr199-phosphorylated PI3K (#4228S, Cell Signaling Technology), Akt (#4691S, Cell Signaling Technology), Ser473-phosphorylated Akt (#4060S, Cell Signaling Technology), LC3B (#2775, Cell Signaling Technology) and β-Actin (#4970S, Cell Signaling Technology). Immunoreactivity was then visualized by probing with a HRP-conjugated secondary antibody (#7074, Cell Signaling Technology) and detected using the ECL kit (#sc-2048, Santa Cruz).

### Immunohistochemical staining

Glioma tissue specimens from mice were fixed overnight with 10%, embedded in paraffin and sectioned at a thickness of 4 µm. Sections depleted of paraffin were incubated with embedding and sectioning. The slides were treated with primary antibody to Ki-67 (#9449S, Cell Signaling Technology) overnight at 4 °C, and then incubated with a HRP-conjugated secondary antibody (#7074, Cell Signaling Technology) at room temperature. Slides were observed under a microscope and tumor cells positive for Ki-67 were counted.

### Cell metabolism measurement

Cellular oxidative phosphorylation and glycolysis were monitored with the Seahorse Bioscience extracellular flux analyzer (XF24, Seahorse Bioscience) by measuring the OCR and ECAR in real time as described previously^39^. Approximately, 10^5^ cells were seeded in specific 24-well plates designed for XF24 in 250 mL of the appropriate growth medium and incubated overnight. Prior to measurements, cells were washed with unbuffered medium once, immersed in 500 mL of unbuffered medium, and incubated in the absence of CO_2_ for 1 h. Three metabolic inhibitors were sequentially loaded at specific time points: oligomycin (inhibititor of ATP synthase, 1 μM), followed by FCCP (a protonophore and uncoupler of mitochondrial oxidative phosphorylation, 0.5 μM), followed by the addition of a combination of rotenone (mitochondrial complex I inhibitor, 100 nM) and myxothiazol (inhibitor of cytochrome C reductase, 100 nM). Basal OCR and ECAR were measured, as well as the changes in oxygen consumption caused by either the treatment or the knock-down of target genes.

### RNAi experiments

Specific siRNAs targeting Syk (#siB09122384015) and PI3K (#siB171206112648) were purchased from RiboBio, China. siRNAs were transfected using Lipofectamine RNAiMAX (Life Technologies) with OPTI-MEM (Life Technologies) according to the manufacturer’s instructions.

### The determination of combination index

The interaction between R406 and TMZ was evaluated by the calculating combination index (CI) derived from Chou-Talalay equations using CalcuSyn software (Biosoft, Cambridge). CI > 1, CI = 1 and CI < 1 indicate antagonism, additive effect and synergism, respectively.

### Subcutaneous and intracranial animal experiments

For the subcutaneous animal model, 4-week old female BALB/c-nu/nu mice were inoculated with 4 × 10^5^ GSC-1 cells at the lower flank. When palpable tumors developed (50 mm^3^), 32 mice were randomly divided into four groups and were injected intraperitoneally with vehicle, R406 (20 mg/kg daily), TMZ (50 mg/kg daily for 5 consecutive days) and R406 combined with TMZ, respectively. Tumor volume was measured every day with a caliper and calculated tumor volumes using the following formula: shortest diameter^2^ × longest diameter × 0.5. When the volume of the tumor reached 1000 mm^3^, the mice were sacrificed. The glioma tissue specimens were dissected and allocated for western blotting and immunohistochemical analysis.

For the orthotopic animal model, 4-week-old female BALB/c-nu/nu mice were anaesthetized and 1 × 10^5^ GSC-1 cells resuspended with with 2% methylcellulose were intracranially injected 1 mm lateral and 2 mm posterior to bregma and 4 mm deep to the surface of the skull using a micro-syringe and 27 G needle. After 3-day implantation, 28 mice were randomly divided into four groups and injected intraperitoneally with vehicle, TMZ (50 mg/kg daily for 5 consecutive days), R406 (20 mg/kg daily) and R406 combined with TMZ respectively. The survival of mice was recorded and the Kaplan-Meier survival curve was generated.

### Statistical analysis

Statistically significantly differences were evaluated with Student’s *t* test (two-tailed, unequal variance) or ANOVA followed by LSD post-hoc test using SPSS 20 statistical software package (IBM). Statistical significance was defined as *p* values of 0.05 or less.

### Data sharing

The key raw data have been uploaded onto the Research Data Deposit public platform (www.researchdata.org.cn), with the approval RDD number of RDDB2019000535.
